# Radiation exposure of breast tissue in lymphoma radiotherapy: a systematic review of breast dose metrics published since 2000

**DOI:** 10.2340/1651-226X.2025.43177

**Published:** 2025-08-26

**Authors:** Hannah C. Chamberlin, Georgios Ntentas, David J. Cutter, Richard Cowan, Sacha Howell, Christina Hague, John Radford, Sue Astley, Eliana Vasquez Osorio, Marianne Aznar

**Affiliations:** a Radiotherapy Related Research Group, Division of Cancer Sciences, University of Manchester, Manchester, UK; b Guy’s and St Thomas’ NHS Foundation Trust, Medical Physics, London, United Kingdom; c University of Oxford, Nuffield Department of Population Health, London, United Kingdom; d King’s College London, School of Biomedical Engineering & Imaging Sciences, London, United Kingdom; e Oxford Cancer and Haematology Centre, Oxford University Hospitals NHS Foundation Trust, Oxford, UK; f Department of Clinical Oncology, The Christie NHS Foundation Trust, Manchester, UK; g Division of Cancer Sciences, University of Manchester, Manchester, UK; h Division of Informatics, Imaging and Data Sciences, Faculty of Biology, Medicine and Health, University of Manchester, Manchester Academic Health Science Centre, Manchester, UK

**Keywords:** Lymphoma, radiotherapy, radiation dosage, systematic review, breast, cancers, radiation induced

## Abstract

**Background and purpose:**

We present a systematic review of breast dose metrics reported in lymphoma patients receiving radiotherapy and provide reporting recommendations for breast dose in future publications.

**Methods and materials:**

Studies reporting breast doses in lymphoma radiotherapy published between January 2000 and May 2023 were included. Frequency of reporting factors likely to affect breast dose were calculated. Doses for the most frequently reported metrics (mean breast dose (MBD) (Gy, percentage of prescription), V5Gy and V10Gy (%)) were calculated across articles and compared for target volume approaches, radiotherapy techniques, and inclusion of the axilla.

**Results:**

Thirty-four distinct breast dose metrics were found across 57 articles. MBD was the most commonly reported. Axilla irradiation significantly increased MBD, V5Gy and V10Gy, yet 21 articles reported breast doses for a mixed cohort with respect to axillary irradiation. Forty-eight of 57 articles did not report the breast contouring guidelines used. Among articles reporting MBD for proton or butterfly-volumetric modulated arc therapy (VMAT), there was no significant reduction in breast radiation dose for protons compared to butterfly-VMAT.

**Interpretation:**

A wide variety of breast dose metrics are reported in the literature, making it challenging to pool breast tissue exposure data in lymphoma radiotherapy. Factors shown in individual studies to affect breast dose should be reported more systematically to enable large scale analysis. Reporting the presence/absence of axillary irradiation is crucial, due to the significant effect on breast dose. We provide reporting recommendations for breast dose metrics to improve research into radiotherapy-induced breast cancer.

## Introduction

Lymphomas are the sixth most common cancer in the UK, with approximately 2,100 individuals diagnosed with Hodgkin lymphoma (HL) and 14,000 for non-Hodgkin lymphoma (NHL) per year [1, 2]. Treatment for HL and NHL often involves a combination of chemotherapy and radiotherapy (RT), with UK 5-year survival rates of over 80% for HL and 65% for NHL [3, 4]. However, treatment is associated with increased risk of late effects, including cardiovascular disease and second primary cancers [5–8], which may be particularly elevated in younger patients.

Breast screening programmes exist in many countries for women treated with RT for HL at a young age due to the greater risk of RT-induced breast cancer (RIBC) [9, 10], with a standardised incidence ratio of 5.0 reported for breast cancer (BC) in women treated under the age of 36 with RT to the chest [5]. The relative risk varies markedly according to age at RT exposure, with a 22-fold risk for women treated with supradiaphragmatic RT aged 10–14 years, compared to a 2.4-fold risk when treated aged 30–35 years [5].

Current guidelines from the International Lymphoma Radiation Oncology Group recommend minimising the radiation dose to breast tissue and provide the following dose constraints: mean breast dose (MBD) < 4Gy [11, 12], and V4Gy < 10% (or V10Gy < 10% if the former cannot be achieved) [11]. However, as evidence continues to gather about the relationships between radiation dose to breast tissue and the risk of RIBC, it remains important to report more dose metrics than just those used for planning and optimisation.

A retrospective study that calculated radiation dose to the location where BC developed following RT found a wide interquartile range of doses (5.8 to 37.2 Gy) [13]. Also, studies in childhood cancer survivors have suggested that in addition to radiation dose to the chest/breast tissue, increased anthracycline dose [14], pelvic/ovarian irradiation [15] and genetic factors [16], affect risk. Thus, the risk of RIBC is multifactorial and risk modelling is currently hindered by a lack of large datasets containing comprehensive records of all relevant risk factors and detailed RT dose distributions. The relationship between the volume of breast tissue irradiated and risk, which requires detailed descriptions of RT dose distribution, has been highlighted as a gap in knowledge needed for risk modelling [17].

Prescribed dose to the tumour (target volume) is a poor surrogate for breast tissue exposure in modern RT, due to the reduced volumes from conformal RT techniques which treat to involved nodes (IN) and involved sites (IS), compared to the mantle fields of the past [18]. In addition, the increase of intensity-modulated approaches (intensity modulated radiotherapy [IMRT] and volumetric modulated arc therapy [VMAT]), has resulted in average metrics, such as MBD, not sufficiently describing complex and individualised RT dose distributions within the breast tissue. Variation in contouring of the breast between clinicians [19] will also affect dose metrics which are relative to the volume of the breast, such as MBD.

Current NHS England guidelines recommend high risk screening for populations at risk in a pragmatic fashion, specifying only age at exposure (10 to 35 years), ‘radiotherapy to sites involving breast tissue’, a time interval since treatment before screening commences [9], and is on the basis that all women who receive RT to breast tissue are at sufficient risk of developing RIBC to warrant screening. Developing models for RIBC risk would be beneficial at two points in the patient pathway; (1) to use the risk of RIBC to inform the RT strategy, such as which techniques to consider or even whether RT should be omitted, and (2) to tailor screening for patients proportional to their risk, that is to avoid potentially unnecessary screening in low-risk women. The International Guideline Harmonization Group currently recommend breast screening for patients who received a prescribed RT dose of 10 Gy or more to the chest [17]. However, recent evidence on RIBC risk after total body irradiation (TBI) suggests lower doses are also associated with increased RIBC risk [20]. Therefore, it will be important to incorporate accurate measures of dose received to breast tissue at multiple dose levels for individualised risk modelling of RIBC, as this varies greatly between patients.

Therefore, to better understand how breast dose is reported in studies of RT for HL/NHL, this systematic review aims to analyse reported breast dose metrics in published studies on RT for lymphoma patients. A secondary aim is to provide recommendations for harmonising reporting of radiation dose to the breast.

## Methods

### Study identification

**Figure 1:**
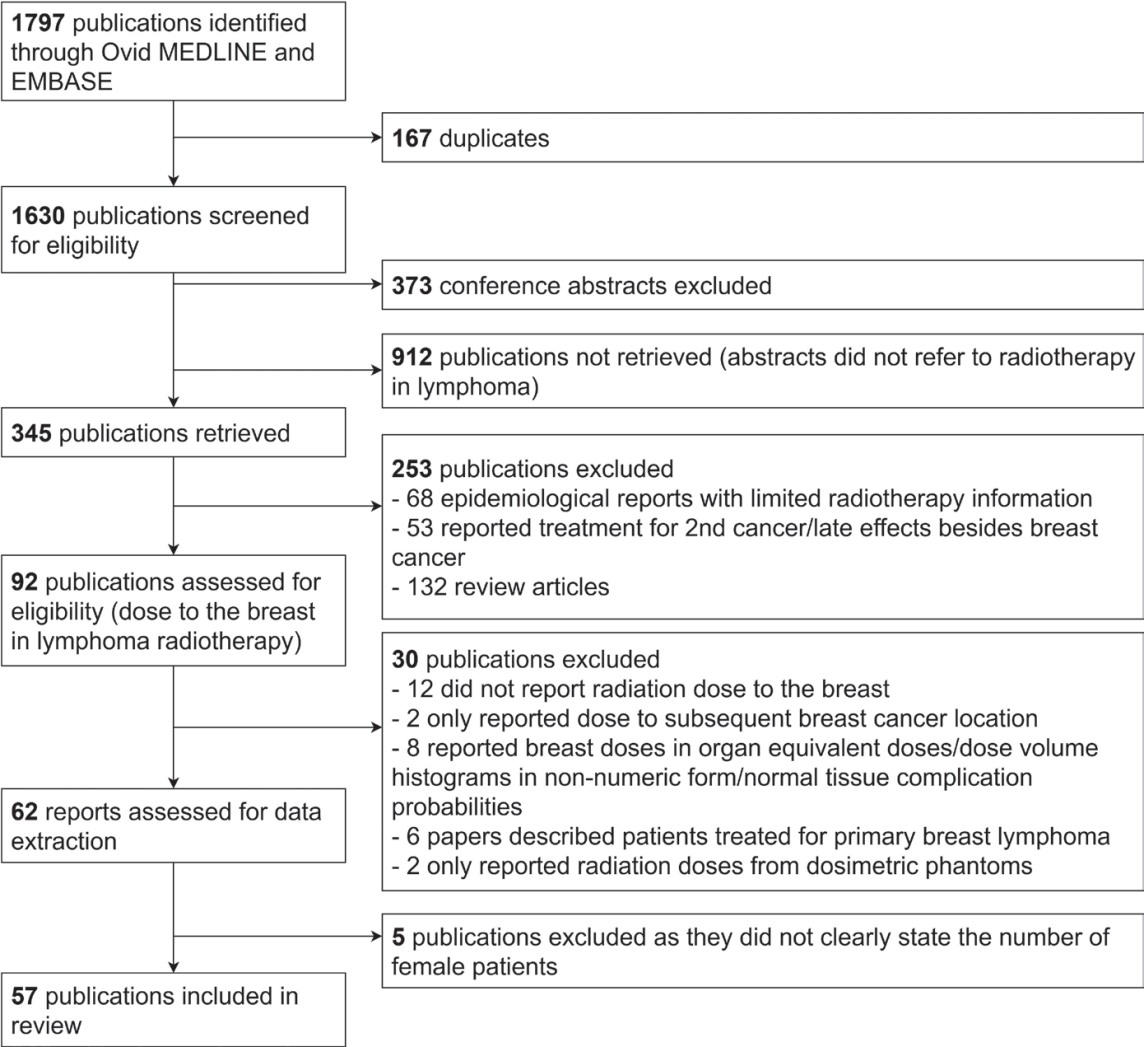
Preferred Reporting Items for Systematic Reviews and Meta-Analyses diagram for studies included in this review.

The Preferred Reporting Items for Systematic Reviews and Meta-Analyses (PRISMA) guidelines were used to identify appropriate studies [21], as shown in Figure 1. The databases Medline and Embase were searched using the terms (lymphoma AND dos* AND [radiation OR radiation therapy OR RT] AND breast AND English language AND year 2000 onwards inclusive) to retrieve articles written in the English language published between 01 January 2000 and 23 May 2023. Studies reporting any measure of breast radiation dose were eligible, regardless of whether the plans were delivered. Studies were then excluded if: RT was not planned on a patient CT (e.g. studies on anthropomorphic phantoms), the dose reported was only estimated at the location of subsequent cancers, or dose to breast tissue was only reported as ‘organ equivalent dose’. Additionally, articles were excluded if the number of female patients was not specified or if the paper reported on a mixed gender cohort and contained insufficient detail to be certain that only female breasts were included.

### Data extraction

The factors retrieved from each paper were; paper year, authors, country of 1st author’s institution, years of patient inclusion, number of female patient plans, total number of patient plans, lymphoma type, target volume approach (i.e. involved node radiotherapy [INRT], etc…), whether breast contouring guidelines were stated, arm positioning, breathing technique, use of an inclined board, mediastinum as target, axilla as target, neck/supraclavicular fossa (SCF) as target, laterality of RT treatment field, RT delivery techniques (i.e. VMAT, IMRT, etc.), treatment delivered, total dose, and fractionation.

The following radiation dose metrics were extracted: MBD, median breast dose, maximum/minimum breast dose, volumetric measures (VxGy = volume irradiated to × Gy or more) and dose level measures (Dx = dose received by ×% or × cm^3^ of the breast volume). We also extracted the breast laterality when mentioned. We define that a paper reported a ‘both breasts’ value if a single dose value was given for the breasts, rather than a value for each breast. Further details can be found in Supplementary Appendix A.

We recorded the number of articles reporting a given metric, as well as the number of regimens and plans within an article. A regimen was defined as a distinct combination of the factors listed above within an article (e.g. in a planning study, each technique evaluated would constitute a unique ‘regimen’), and the dose metrics reported per regimen were recorded. A plan was defined as a RT plan planned on the CT of a female patient, and we recorded the number of plans per regimen. When the same patient was re-planned on a different regimen, that patient was counted in both regimens, thus number of plans does not equate to number of patients.

### Data analysis

Data analysis was conducted on the variables listed above and in Supplementary Appendix A.

We focused dose metrics analysis on MBD (Gy), V5Gy and V10Gy (%) as these metrics were the three most commonly reported. Summary statistics for these dose metrics can be seen in Table 1. We compared doses reported to both breasts only. To increase the number of values reported to both breasts, we calculated the mean of the left and right breast doses from articles which reported these, to give a representative value for the dose reported to both breasts. This was motivated by a paper reporting dose to ‘left’, ‘right’ and ‘both’ breast(s), where the mean of left and right values was equivalent to the ‘both breasts’ value [22]. These values, including those extracted from articles and calculated from articles, were pooled and named ‘both calculated’ metrics: MBD_both (calc)_ (Gy), V5Gy_both (calc)_ (%) and V10Gy_both (calc)_ (%). We also calculated MBD as a percentage of the prescription RT dose (MBD_both (calc)_ (%pres)), when prescription dose was stated and a single value was given per regimen, as detailed in Table 1.

**Table 1:** The frequency of reporting of the MBD (Gy), V5Gy and V10Gy (%) metrics recorded in all articles for left, right and both breasts.

Metric	Number of articles	Number of regimens	Mean	Median (Range)
MBD both (Gy)*	21	101	4.0	2.8 (0.1–20.1)
MBD left (Gy)	23	96	3.3	2.9 (0.1–14.0)
MBD right (Gy)	23	96	3.2	1.9 (0.0–15.9)
MBD_both (calc)_ (Gy)^†^	43	192	3.5	2.5 (0.1–20.1)
MBD_both (calc)_ (%pres) **	39**	179	12.2	8.7 (0.0–65.7)
V5Gy both (%)*	6	19	27.2	26.0 (8.0–54.0)
V5Gy left (%)	11	46	11.7	7.2 (0.0–61.3)
V5Gy right (%)	11	46	15.0	5.3 (0.0–60.6)
V5Gy_both (calc)_ (%)^†^	16	63	17.2	13.0 (0.0–58.5)
V10Gy both (%)*	9	35	10.2	5.7 (0.6–46.0)
V10Gy left (%)	7	20	11.4	11.3 (2.5–21.1)
V10Gy right (%)	6	17	7.6	5.5 (0.9–28.9)
V10Gy_both (calc)_ (%)^†^	16	55	10.2	8.0 (0.6–46.0)

The both calculated (both (calc)) values for each metric are also stated, and the mean, median and range of all metrics are given. The summary for the calculated value of both breasts MBD as a percentage of prescription dose (MBD_both (calc)_ (%pres)) is also given. Similar analysis for V4Gy and V20Gy (%) is reported in the Supplementary Appendix (Table A3). Gy = Gray, MBD = mean breast dose, V5Gy (%) = percentage volume of breast tissue receiving at least 5 Gray, V10Gy (%) = percentage volume of breast tissue receiving at least 10 Gray, %pres = percentage of prescription dose.

* We define ‘both breasts’ for articles which defined a single dose value for the breasts, rather than 2 individual values for the left and right breasts.

** The number of papers which reported both mean breast dose and gave a single value for the prescription dose per regimen, therefore enabling us to calculate MBD_both(calc)_ (%pres).

† For articles reporting left and right breast doses individually, the mean of the 2 values were calculated to give a representative value for the dose to both breasts, which we name ‘both (calc)’. Thus, the MBD_both(calc)_ (Gy) from 43 articles (21+23-1 articles, as 1 article reported MBD for left, right and both breasts. The both breasts value was equivalent to the mean of the values in the individual breasts), V5Gy_both(calc)_ (%) from 16 articles (11+6-1 article, as 1 article reported V5Gy (%) for left, right and both breasts) and V10Gy_both(calc)_ (%) from 16 articles (the article for which only left V10Gy (%) was reported was included and those values were taken as the combined V10Gy_both(calc)_ (%)) were included for analysis.

Similar analysis for V4Gy_both (calc)_ (%) and V20Gy_both (calc)_ (%) is detailed in Supplementary Table A3 to provide additional detail at lower and higher doses.

Some regimens were removed from analysis due to mixed grouping for a given variable, such as where one MBD value was given for a group of patients treated in a mixture of arm positions. Regimens were additionally removed if there was ambiguity in a variable, such as when the RT delivery technique was only stated as ‘photon’.

Mann–Whitney *U* tests were conducted to test for significant differences in a breast dose metric for different conditions of an impacting variable (i.e. arm position up or down). Data analysis was conducted in R (version 4.1.3) using package *forestploter* [23].

## Results

Fifty-seven articles fulfilled the inclusion criteria (see Figure 1).

Forty-eight out of 57 articles included only HL patients, totalling 2033 plans on female patients. The remaining 9 articles reported on mixed HL and NHL cohorts, totalling 360 plans on female patients.

### Frequency of reported breast dose metrics

Thirty-four different radiation dose metrics, irrespective of laterality, were recorded (Supplementary Table A1). When considering breast laterality, 83 different dose metrics were reported across the 57 included articles (Supplementary Table A2).

MBD was the most commonly reported breast dose metric, with 23 studies reporting MBD for left and right breasts, and 21 reporting MBD to both breasts (Supplementary Table A2). The most common VxGy measure was V5Gy (%) (reported 6, 11 and 11 times for both, left and right breasts, respectively), while the most common Dx measure was D1% (reported 4, 3 and 3 times for both, left and right breasts, respectively).

All Dx metrics identified were reported in terms of percentage of breast volume, rather than absolute volume. Similarly, 55/57 articles reported VxGy as percentage of breast volume.

### Breast contouring approach

The majority of studies (48/57) did not report the guidelines used for contouring breast tissue. Four studies reported contouring the breast using the fibroglandular tissue visible within a fixed range of Hounsfield units (HU) as guidance [24–27]. Three articles used guidelines set out by White et al. [28] and another 2 articles described their methods, either as ‘the extent of glandular tissue’ [29] or by detailing the anatomical borders used [30].

### Target volume approach

**Figure 2:**
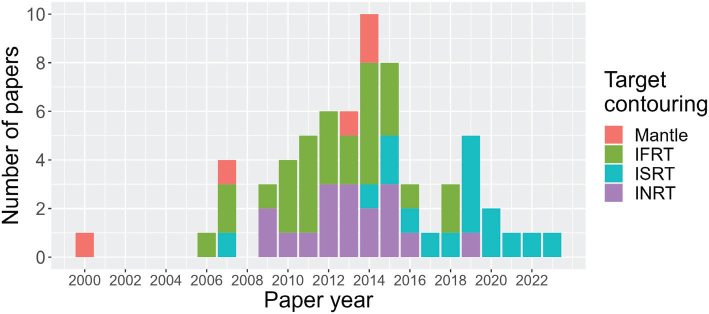
The frequency of target volume approaches in the year of article publication. IFRT: involved field radiotherapy, ISRT: involved site radiotherapy, INRT: involved node radiotherapy.

All but 2 articles reported their target volume approach (Supplementary Table A4). There was an increasing proportion of articles reporting an INRT or ISRT approach over time (Figure 2).

**Figure 3:**
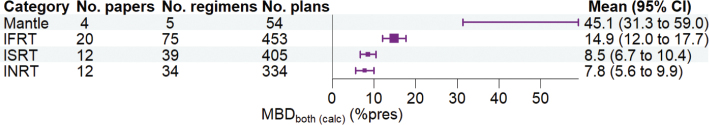
Forest plot of the mean breast dose as a percentage of the prescribed dose (MBD_both (calc)_ (%pres)) for the target volume techniques of mantle radiotherapy, IFRT (involved field radiotherapy), ISRT (involved site radiotherapy) and INRT (involved node radiotherapy).

MBD_both (calc)_ (%pres) was only compared where the same target volume approach was used for all plans within a regimen (Figure 3). As expected, MBD_both (calc)_ (%pres) was greatest for mantle (45.1% [95% CI 31.3 to 59.0%]), followed by IFRT (14.9% [95% CI 12.0 to 17.7%]), which was significantly lower (*p* < 0.01). MBD_both (calc)_ (%pres) for IFRT was significantly greater than ISRT (8.5% [95% CI (6.7 to 10.4)]) (*p* < 0.01).

Few articles reported V5Gy and V10Gy (%) doses for IN and IS (Supplementary Table A5).

We intended to report target position relative to the breasts as this will influence breast dose, but this was not often reported.

### Mediastinum and axilla

Fifty articles included patients treated to the mediastinum, 27 of which reported radiation dose for patients treated only to the mediastinum (Supplementary Table A6). Eight articles reported breast doses for patients treated with axillary and mediastinal irradiation. Twenty-one articles reported breast dose metrics for mixed cohorts of patients with respect to axillary irradiation.

**Figure 4:**
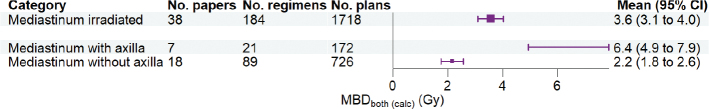
Calculated combined mean breast dose in Gray (MBD_both (calc)_ (Gy)) calculated for regimens which did not specify axillary irradiation (mediastinum irradiated), regimens which reported MBD for patients treated to the mediastinum and axilla, and regimens for which patients were treated to the mediastinum only.

Irradiating both the mediastinum and axilla significantly increased MBD_both (calc)_ (Gy) (see Figure 4) compared to irradiating the mediastinum alone; 6.4 Gy (95% CI 4.9 to 7.9) vs. 2.2 Gy (95% CI 1.8 to 2.6) (*p* < 0.01). Similar significant trends were seen for MBD_both (calc)_ (%pres), V10Gy_both (calc)_ and V5Gy_both (calc)_ (*p* < 0.01 for all pairwise comparisons, except *p* = 0.11 for V10Gy_both (calc)_ (%) for mediastinum (regardless of axilla) compared to mediastinum without axilla) (Supplementary Table A7).

### Radiotherapy technique

The most frequently reported RT delivery technique was three-dimensional conformal radiotherapy (3DCRT) (39 articles), followed by IMRT (20 articles) and volume modulated arc radiotherapy (VMAT) techniques (16 articles reported values for VMAT, and 6 articles for butterfly-VMAT [BVMAT]) (Supplementary Table A8).

**Figure 5:**
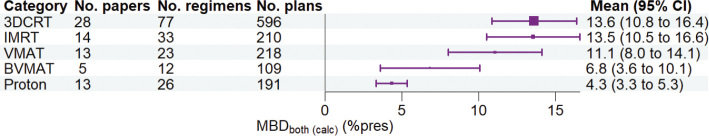
Calculated combined mean breast dose as a percentage of prescribed dose (MBD_both (calc)_ (%pres)) calculated across articles for given radiotherapy delivery techniques. 3DCRT: three-dimensional conformal radiotherapy. IMRT: intensity modulated radiotherapy. VMAT: volumetric modulated arc therapy. BVMAT: butterfly-volumetric modulated arc therapy

For MBD_both (calc)_ (%pres) (Figure 5), 3DCRT and IMRT were similar (13.6% and 13.5%, respectively, *p* = 0.44). VMAT (11.1%) was not significantly less than 3DCRT (*p* = 0.76) or IMRT (*p* = 0.28). However, BVMAT (6.8%) was significantly less than 3DCRT (*p* = 0.04), and IMRT (*p* = 0.02). Protons (4.3%) were significantly less than VMAT (*p* < 0.01) but not BVMAT (*p* = 0.28).

In contrast to MBD_both (calc)_ (%pres), 3DCRT was significantly lower than IMRT for V5Gy_both (calc)_ (%) (*p* = 0.01) (Supplementary Table A9). Infrequent reporting limited the conclusions which could be drawn between other techniques and at different metrics.

Further analysis on the effect of breast dose due to the neck/SCF inclusion, breathing technique, position on table, and arm position can be found in the Supplementary Appendix.

## Discussion

This is the first systematic review of breast doses reported in modern RT to treat lymphomas. This study analysed 57 articles, totalling 2,393 RT plans for female patients. The findings highlight the need for consistent reporting of breast dose metrics and other clinical details which influence breast dose, particularly axillary involvement.

The rationale behind reporting at the given dose levels is rarely stated. A matched case-control analysis of relative BC risk following RT and chemotherapy for HL suggested risk was only significantly increased in patients who receive ≥ 4 Gy to the second primary BC location [7]. This finding has undoubtedly encouraged the reporting of V4Gy in subsequent studies, but is not a reason to consider it the most relevant dose-volume metric to report. Russell et al. found an interquartile range in dose received to the subsequent BC location of 5.8–37.2 Gy following RT for lymphoma [13], and doses up to 61.3 Gy were reported to the site of second BC by Travis et al. [7], suggesting both low and high doses are critical to report. A recent case-control study concluded that MBD predicts BC risk in HL patients, but only analysed patients treated prior to 2000, thus no patients with IS or IN RT, or with techniques, which cause low dose baths such as BVMAT [31]. Therefore, it is likely important to report more breast dose metrics across a range of doses until RIBC risk has been assessed with modern treatment techniques.

The majority of articles analysed did not mention the breast contouring technique; those which did used a variety of methods. Two articles followed guidelines developed for BC patients, where breast tissue is the target volume [28, 32]. In a paper pre-dating contouring guidelines for BC (‘target breast contouring’), contouring variation in the cranial region of the breast was found to have an average inter-observer deviation of 28 mm [19]. Reducing the cranial contouring variation with breast as an organ-at-risk contouring guidelines will aid reliably calculating breast radiation dose for lymphoma patients with a target high in the chest.

While the majority of articles stated their target volume approach, and we found MBD from INRT/ISRT was significantly lower than for IFRT, we could not account for target size and position, which is likely a strong contributor to breast dose.

MBD to patients treated to both the mediastinum and axilla was significantly greater than MBD for patients treated to the mediastinum alone. This is important as the greatest proportion of BC locations following RT for HL have been reported in the upper outer quadrant [13]. However, this represents the most common site for BC in the general population [33] and the extent to which risk is increased by axillary irradiation has yet to be determined. This is also important because the axilla is not commonly involved in early-stage HL (e.g. < 10% in PET negative patients treated with IFRT in the RAPID trial [34]). We hoped to analyse the effect of breast dose with respect to inclusion of the neck/SCF, as these fields could result in dose to the superior portion of the breast, but the irradiation of these fields was infrequently reported.

Our analysis suggests more modern RT techniques may reduce MBD, such as BVMAT and proton RT. We were unable to see similar trends with V5Gy and V10Gy due to infrequent reporting. Analysing multiple dose metrics with respect to RT technique is important, as although it is assumed that the relationship between radiation exposure of breast tissue and BC risk is linear with no threshold [7, 35], there remains uncertainty if the same integral dose delivered in different ways will result in the same RIBC risk (e.g. the same MBD with a low dose to a large volume versus a high dose to a smaller volume).

We found great inconsistency in reporting dose to separate breasts (left and right) or dose to a structure combining both breasts. To mitigate this in our analysis, we estimated a ‘both calculated’ breast dose for articles reporting dose to separate breasts, by taking the average of the left and right breast values. This has limitations, for example, for a very one-sided RT plan. If one breast received 0 Gy, this method halves the dose for the breast in the RT field, thus gives a skewed measure of breast dose.

We noted only 2 articles out of 57 reporting × Gy in absolute volumes (cm^3^) as opposed to percentage. To develop personalised estimates of RIBC risk, we believe recording patients’ absolute volume at risk will be important, as percentage volume measurements are influenced by breast volume. In cohorts with mixed diagnoses (and therefore a range of prescription RT doses), it may also help to provide breast tissue exposure data in Gy, in addition to percentage of prescribed dose commonly used in planning, to facilitate later modelling efforts.

Improvements in accurate breast dose estimates, alongside recording of other BC risk factors, will aid development of more accurate risk models, thus personalised breast screening in this population. Reporting a range of dose metrics, including high and low doses to describe the complex dose distributions in modern RT techniques, will be an important interim approach while sharing full dose distributions in multi-centre studies remains challenging. In addition, reporting patient and technical set-ups found to affect breast dose (such as breath hold, arm positioning, and position on table) could provide further information [36–38].

We make the following recommendations for reporting breast dose metrics to aid research on RIBC risk in young women treated for lymphoma.

To more accurately estimate breast dose:

1. Use recognised breast contouring guidelines and clearly describe the guidelines in publications.
2. Report radiation dose to left and right breasts separately, as well as dose to a combined breast structure, to most accurately describe the dose distribution, while enabling comparison with previous studies.
3. Until the minimum number of parameters needed to quantitatively describe breast dose and associated RIBC risk in modern techniques has been established, report MBD and the volume of breast tissue irradiated in both absolute and percentage breast volumes, to at least V5Gy and V10Gy, with additional dose metrics of V2Gy, V4Gy, V15Gy, V20Gy and V30Gy recommended. This will help multi-study analysis of the risk of RIBC at different doses. Creating a research database of RT plans would be the gold standard for this research. This should span many ages at treatment, as this analysis could further refine RIBC risk models based on age at treatment, and the planning constraints that should be used for individual women.
4. Make clear if the RT field included the axilla, and only report breast dose metrics for groups if patients within the group have the same axillary fields (considering laterality).

To improve reporting of factors that likely influence breast dose, thus inform future RT planning strategies:

1. Report the target volume approach used in RT treatment planning. In addition, report separate breast dose metrics for each approach, in the case of cohorts treated with mixed approaches (e.g. treated with IFRT and INRT).
2. Report on patient and technical set-up factors, which have been found in individual studies to impact breast dose, for example RT delivery technique, position on the table, arm positioning, and breathing technique.
3. Report the position of the target relative to the breast volume.

## Conclusion

In this systematic review, we have demonstrated the need for greater standardisation in reporting dose to breast tissue in lymphoma RT, especially factors likely to have a strong impact on breast dose, such as target volume approach and presence/absence of axillary irradiation.

More systematic reporting of breast dose in research articles will improve quantitative description of RT plans without access to the planning CT, enabling greater comparisons across patient cohorts. We propose to report a wide range of breast dose metrics until further research clarifies the doses which increase the risk of RT-induced BC in modern RT treatments.

## Supplementary Material



## Data Availability

This article analyses data published in the literature. As a result, data sharing is not applicable to this article as no new datasets were generated or analysed during this study.
