# Supplementary material for: Radiation exposure of breast tissue in lymphoma radiotherapy: a systematic review of breast dose metrics published since 2000
Source: Acta Oncol. 2025 Aug 26;64:43177. doi: 10.2340/1651-226X.2025.43177 (PMC12398104; doi:10.2340/1651-226X.2025.43177)
Supplement: Supplementary file 1 [file AO-64-43177-s1.pdf]

Supplementary material has been published as submitted. It has not been copyedited, or typeset by Acta Oncologica

Appendix A

Data Analysis

Data extraction details

Data extracted from each paper, if included in the paper, was:

- Paper year
- Country of 1st author's institution
- Years of patient inclusion
- The use of breast contouring guidelines
- Number of female patients
- Lymphoma type (Hodgkin lymphoma or non-Hodgkin lymphoma)
- Age information stated in paper (typically age ranges, or individual ages if reporting dose per patient plan)
- Target contouring approach (mantle, involved field (IF), involved site (IS), involved node (IN))
- Arm positioning (both arms up, arms akimbo, arms down)
- Use of breathing adaptation (deep inspiration breath hold (DIBH) compared to free breathing (FB))
- Position on treatment table (flat, inclined board or prone)
- The region irradiated (noting mention of mediastinum, axilla and neck/SCF)
- Stage of cancer (typically a range of values given, or the individuals' staging if individuals plans described)
- Laterality of the lymphoma/radiotherapy field
- Radiotherapy delivery technique (3D conformal radiotherapy (3DCRT), intensity modulated therapy (IMRT), volumetric arc radiotherapy (VMAT), butterfly volumetric arc radiotherapy (BVMAT), proton)
- Whether the radiotherapy was delivered or just planned
- The delivered dose
- The delivered fractions
- The fraction dose

Dose metrics collected are detailed in table A2.

Frequency of reported breast dose metrics

Table A1: Frequency of papers which reported a given dose metric for any breast structure (i.e. either both breasts or left/right breasts individually). Units; cm^3: centimetres cubed (an absolute volume measure), %: percentage of breast volume (a percentage volume measure).

Overall breast dose metrics	Number of papers reporting
Mean	42
V10Gy (%)	17
V5Gy (%)	16
V4Gy (%), V20Gy (%)	14
Maximum (Gy)	11
V30Gy (%)	10
V15Gy (%)	7
D1pc (Gy)	6
V25Gy (%), Minimum (Gy)	5
D50pc (Gy), Volume (cm^3)	4
Median of mean (Gy), Median (Gy)	3
V5Gy (cm^3), D70pc (Gy)	2
V20Gy (cm^3), V30Gy (cm^3), D2pc (Gy), D33pc (Gy), V1Gy (%),V1Gy (cm^3), V3Gy (%), V16Gy (%), V24Gy (%), V1.5Gy (%), V2Gy (%), V4.5Gy (%), V7.5Gy (%), V10.5Gy (%), V15.8Gy (%), V21Gy (%), V3.5Gy (%)	1

Table A2: Frequency of papers which reported a dose metric with specificity for laterality (i.e. specified the left or right breast, or specified that both breasts were included).

Breast dose metric	Number of papers reporting
Left mean dose (Gy), Right mean dose (Gy)	23
Both breasts mean dose (Gy)	21
Left V5Gy (%), Right V5Gy (%)	11
Both breasts V10Gy (%)	9
Left V20Gy (%)	8
Both breasts V4Gy (%), Left V10Gy (%), Right V20Gy (%)	7

Both breasts mean of maximum dose (Gy), Both breasts V5Gy (%), Left V4Gy (%), Left V30Gy (%), Right V4Gy (%), Right V10Gy (%)	6
Both breasts V20Gy (%), Both breasts V30Gy (%), Left maximum dose (Gy), Left V15Gy (%), Right maximum dose (Gy), Right V30Gy (%)	5
Both breasts D1% (Gy), Left V25Gy (%), Right V15Gy (%), Right V25Gy (%)	4
Both breasts volume (cm^3), Both breasts median of mean dose (Gy), Both breasts mean of minimum dose (Gy), Both breasts D50% (Gy), Both breasts V15Gy (%),Left D1% (Gy), Right D1% (Gy)	3
Both breasts D70% (Gy), Both breasts V25Gy (%), Left breast volume (cm^3), Left minimum dose (Gy), Left median dose (Gy), Right breast volume (cm^3), Right minimum dose (Gy), Right median dose (Gy)	2
Both breasts median (Gy), Both breasts D2% (Gy), Both breasts D33% (Gy), Both breasts volume to 50% of maximum dose (%), Both breasts V25Gy (median) (%), Both breasts V1Gy (cm^3), Both breasts V3Gy (%), Both breasts V5Gy (cm^3), Both breasts V16Gy (%), Both breasts V24Gy (%), Left mean dose (% of prescription), Left median dose (% of prescription), Left V1Gy (%), Left V1.5Gy (%), Left V2Gy (%), Left V4.5Gy (%), Left V7.5Gy (%),Left V10.5Gy (%), Left V21Gy (%), Left V5Gy (cm^3), Left V20Gy (cm^3), Left V30Gy (cm^3), Right mean dose (% of prescription), Right median dose (% of prescription), Right V1Gy (%), Right V1.5Gy (%), Right V2Gy (%), Right V4.5Gy (%), Right V7.5Gy (%), Right V10.5Gy (%), Right V21Gy (%), Right V5Gy (cm^3), Right V20Gy (cm^3), Right V30Gy (cm^3), Ipsilateral mean dose (Gy), Ipsilateral maximum dose (Gy), Ipsilateral minimum dose (Gy), Ipsilateral V4Gy (%), Contralateral mean dose (Gy), Contralateral maximum dose (Gy), Contralateral minimum dose (Gy), Contralateral V4Gy (%)	1

V4 and V20 analysis

Table A3; The frequency of reporting of the V4Gy and V20Gy (%) metrics recorded in all papers for left, right and both breasts. The both calculated ('both (calc)') values for each metric are also stated, and the mean, median and range of all metrics are given. Abbreviations: Gy: Gray, MBD: mean breast dose, V4Gy (%): percentage volume of breast tissue receiving at least 4 Gray, V20Gy (%): percentage volume of breast tissue receiving at least 20 Gray.

**Metric**	**No. papers**	**No. regimens**	**Mean**	**Median**	**Min**	**Max**
V4Gy both (%)*	7	21	15.9	8.1	3.5	76.0
V4Gy left (%)	6	18	26.8	19.9	3.4	61.8
V4Gy right (%)	6	18	24.0	15.0	6.0	58.0
$V4Gy_{both\ (calc)}$ (%) †	13	39	20.3	14.6	3.5	76.0
V20Gy both (%)*	6	17	7.2	6.0	0.1	21.2
V20Gy left (%)	8	38	3.4	2.095	0	14.6
V20Gy right (%)	7	35	5.8	0.9	0	43.7
$V20Gy_{both\ (calc)}$ (%) †	13	53	5.3	3.5	0	25.55

*we define "both breasts" for papers which defined a single dose value for the breasts, rather than 2 individual values for the left and right breasts.

†For papers reporting left and right breast doses individually, the mean of the 2 values were calculated to give a representative value for the dose to both breasts, which we name 'both (calc)'. Thus the $V4Gy_{both\ (calc)}$ (%) from 13 papers (7 reporting for both, 6 reporting for the individual breast) and $V20Gy_{both\ (calc)}$ (%) from 13 papers (1 paper reported for the left, right and both breasts, and the both breasts value was used for the both (calc) value). The paper for which only left V20Gy (%) was reported was included and those values were taken as the combined $V20Gy_{both\ (calc)}$ (%).

Target contouring

Table A4. Numbers of papers, regimens and plans which report on the specified target contouring. Papers may report multiple regimens, thus the number of papers does not add to the number of papers included in the systematic review. Abbreviations; IFRT: involved field radiotherapy, ISRT: involved site radiotherapy, INRT: involved node radiotherapy, N.S.: not stated.

Target contouring	Number of papers	Number of regimens	Number of plans
Mantle	5	11	114
IFRT	27	91	597
IFRT/ISRT/INRT	1	4	56
IFRT/ISRT	1	3	42
IFRT/INRT	1	2	32
ISRT	16	54	797
ISRT/INRT	2	18	66
INRT	17	46	477
Residual volume	1	1	10
N.S.	2	4	202

Table A5: Calculated combined mean breast dose in Gray ($MBD_{both\ (calc)}$ (Gy)) and calculated combined percentage volume of breast ($VxGy_{both\ (calc)}$ (%)) receiving at least 4, 5 10 and 20Gy calculated across papers for given target contouring techniques. Note that the number of papers, regimens and plans included here are different compared to figure 3 in the main text, as some papers may not have reported the prescribed dose/not given a single value for it. Abbreviations: IFRT: involved field radiotherapy, ISRT: involved site radiotherapy, INRT: involved node radiotherapy.

Dose metric	Category	No. papers	No. regimens	No. plans	Mean (95% CI)
$MBD_{both\ (calc)}$ (Gy)	Mantle	4	5	54	13.3 (9.3 to 17.2)
	IFRT	22	79	469	4.0 (3.4 to 4.7)
	ISRT	13	45	705	2.7 (2.0 to 3.3)
	INRT	12	33	335	2.7 (2.0 to 3.3)
$V4Gy_{both\ (calc)}$ (%)	IFRT	4	9	84	32.2 (15.7 to 48.7)
	ISRT	2	6	90	14.5 (9.9 to 19.1)
	INRT	5	17	161	19.0 (11.6 to 26.4)
$V5Gy_{both\ (calc)}$ (%)	Mantle	2	7	68	36.8 (25.3 to 48.3)
	IFRT	9	20	161	15.3 (10.2 to 20.3)
	ISRT	4	8	39	11.3 (3.4 to 19.3)
	INRT	3	5	56	12.6 (1.5 to 23.7)
$V10Gy_{both\ (calc)}$ (%)	Mantle	1	6	60	29.3 (17.4 to 41.3)
	IFRT	7	17	186	9.8 (7.4 to 12.1)
	ISRT	3	9	71	4.2 (1.7 to 6.8)
	INRT	6	18	171	7.2 (3.9 to 10.4)
$V20Gy_{both\ (calc)}$ (%)	IFRT	7	16	123	7.6 (3.6 to 11.6)
	ISRT	3	6	31	2.1 (0.7 to 3.5)
	INRT	4	11	110	5.1 (1.9 to 8.3)

Mediastinum and axilla

Table A6: Frequency of reporting the areas of the chest irradiated. Abbreviations: N.S.: not stated.

**Subgroup**	**Categories**	**Number of papers**	**Number of regimens**	**Number of plans**
Mediastinum targeted				
	Yes	50	221	2255
	Some patients	6	11	128
	N.S.	1	2	10
Axilla targeted				
	Yes	8	27	232
	Some patients	21	69	727
	No	27	115	1014
	N.S.	8	23	420

Table A7: Calculated combined mean breast dose as a percentage of prescribed dose ($MBD_{both\ (calc)}$ (%pres)) and calculated combined percentage volume of breast ($VxGy_{both\ (calc)}$ (%)) receiving at least 4, 5, 10 and 20 Gray calculated across papers when stating if the mediastinum was irradiated, and whether the mediastinum was irradiated without the axilla. Note that no more than 1 regimen recorded $V4Gy_{both\ (calc)}$ (%) for patients treated to the mediastinum with axilla, hence no data is reported.

Dose metric	Category	No. papers	No. regimens	No. plans	Mean (95% CI)
$MBD_{both\ (calc)}$ (%pres)	Mediastinum irradiated	33	168	1318	12.4 (10.7 to 14.2)
	Mediastinum with axilla	5	17	70	24.1 (17.0 to 31.1)
	Mediastinum without axilla	16	84	583	7.4 (5.6 to 9.1
$V4Gy_{both\ (calc)}$ (%)	Mediastinum irradiated	11	34	444	19.4 (14.1 to 24.7)
	Mediastinum without axilla	7	22	269	15.1 (9.2 to 21.0)
$V5Gy_{both\ (calc)}$ (%)	Mediastinum irradiated	15	61	548	17.4 (13.3 to 21.4)
	Mediastinum with axilla	2	11	65	38.4 (30.1 to 46.8)
	Mediastinum without axilla	8	36	235	8.1 (5.3 to 10.9)
$V10Gy_{both\ (calc)}$ (%)	Mediastinum irradiated	14	50	543	10.3 (7.4 to 13.2)
	Mediastinum with axilla	1	6	60	29.3 (17.4 to 41.3)
	Mediastinum without axilla	10	36	408	6.5 (4.7 to 8.4)
$V20Gy_{both\ (calc)}$ (%)	Mediastinum irradiated	12	51	322	5.5 (3.7 to 7.2)
	Mediastinum with axilla	1	5	5	13.3 (5.8 to 20.7)
	Mediastinum without axilla	7	35	207	2.9 (1.6 to 4.2)

Radiotherapy technique

Table A8: Frequency table of the planning techniques described across all regimens reported in the papers analysed in this systematic review. Abbreviations: AP: anterior-posterior, PA: posterior- anterior, 3DCRT: three-dimensional conformal radiotherapy, IMRT: intensity modulated radiotherapy, VMAT: volumetric arc radiotherapy, BVMAT: butterfly volumetric arc radiotherapy, N.S.: not stated.

**Planning technique**	**Number of papers**	**Number of regimens**	**Number of plans**
3DCRT	39	95	746
IMRT	20	42	305
VMAT	16	27	253
BVMAT	6	14	139
Photon	4	9	282
Proton	17	33	384
Multiple	1	1	18
N.S.	1	2	152

Table A9: Calculated combined mean breast dose in gray ($MBD_{both\ (calc)}$ (Gy)) and calculated combined percentage volume of breast ($VxGy_{both\ (calc)}$ (%)) receiving at least 4, 5, 10 and 20 Gray calculated across papers for given radiotherapy delivery techniques. Abbreviations: 3DCRT: three-dimensional conformal radiotherapy, IMRT: intensity modulated radiotherapy, VMAT: volumetric arc radiotherapy, BVMAT: butterfly volumetric arc radiotherapy.

Dose metric	Category	No. papers	No. regimens	No. plans	Mean (95% CI)
$MBD_{both\ (calc)}$ (Gy)	3DCRT	29	78	595	3.7 (3.1 to 4.4)
	IMRT	14	34	229	4.1 (3.3 to 4.9)
	VMAT	14	24	219	3.9 (2.6 to 5.1)
	BVMAT	5	12	109	2.0 (1.0 to 3.0)
	Proton	15	31	367	1.4 (1.0 to 1.7)
$V4Gy_{both\ (calc)}$ (%)	3DCRT	9	12	130	17.5 (6.5 to 28.5)
	IMRT	6	10	97	36.7 (25.9 to 47.5)
	VMAT	3	5	64	15.7 (5.3 to 26.2)
	BVMAT	4	6	95	10.8 (5.1 to 16.4)
	Proton	4	4	41	13.4 (7.3 to 19.6)
$V5Gy_{both\ (calc)}$ (%)	3DCRT	11	27	174	10.9 (6.6 to 15.1)
	IMRT	6	13	69	25.3 (15.6 to 34.9)

	VMAT	3	4	7	14.0 (-2.9 to 30.8)
	BVMAT	1	4	4	11.2 (-3.4 to 25.8)
	Proton	2	4	30	7.7 (1.7 to 13.6)
$V10Gy_{both\ (calc)}$ (%)	3DCRT	11	15	147	8.9 (5.3 to 12.6)
	IMRT	8	12	112	9.8 (6.6 to 13.1)
	VMAT	7	11	109	6.0 (2.4 to 9.5)
	BVMAT	3	4	65	1.8 (0.5 to 3.0)
	Proton	4	5	33	7.7 (1.7 to 13.6)
$V20Gy_{both\ (calc)}$ (%)	3DCRT	11	22	138	6.6 (3.5 to 9.7)
	IMRT	8	14	65	6.4 (3.0 to 9.8)
	VMAT	4	6	37	0.8 (-0.6 to 2.1)
	BVMAT	2	5	14	2.0 (-1.2 to 5.2)

Neck/SCF

We assumed any papers stating neck irradiation would include the SCF within their neck field. Thus we analysed the mention or either neck or SCF within the irradiated region.

As only 1 paper reported MBD for patients not treated to the neck, and there was limited reporting at other dose metrics, the large uncertainty on the values meant no conclusions could be drawn.

Table A10: Frequency of reporting whether the neck or SCF was irradiated. Abbreviations: SCF: supraclavicular fossa, N.S.: not stated.

**Subgroup**	**Categories**	**Number of papers**	**Number of regimens**	**Number of plans**
SCF/Neck targeted	Yes	12	60	624
	Some patients	35	135	1170
	No	1	4	4
	N.S.	10	35	595

Table A11: Calculated combined mean breast dose in gray ($MBD_{both\ (calc)}$ (Gy)) and as a percentage of prescribed dose ($MBD_{both\ (calc)}$ (%pres)) calculated across papers when stating if the neck or SCF was irradiated. Abbreviations: SCF: supraclavicular fossa.

Dose metric	Category	No. papers	No. regimens	No. plans	Mean (95% CI)
$MBD_{both\ (calc)}$ (Gy)	Neck/SCF included	9	47	508	4.06 (2.89 to 5.22)
	Neck/SCF not included	1	4	4	6.32 (0.77 to 11.87)
$MBD_{both\ (calc)}$ (%pres)	Neck/SCF included	7	39	206	15.21 (10.48 to 19.94)
	Neck/SCF not included	1	4	4	30.11 (3.69 to 56.52)

Arm positioning

Significant differences in doses between arms up, akimbo and down were not seen for any dose metric. However, this may have been due to the small numbers of studies reporting arm position.

Table A12. Frequency of reporting the arm position when reporting on dose to the breast in lymphoma radiotherapy.

**Arm positioning**	**Number of papers**	**Number of regimens**	**Number of plans**
Up	8	18	127
Up or down	5	37	200
Down	10	39	457
Down or akimbo	3	8	98
Akimbo	3	6	99
N.S.	30	126	1412

Table A13: Calculated combined mean breast dose in gray ($MBD_{both\ (calc)}$ (Gy)), as a percentage of prescribed dose ($MBD_{both\ (calc)}$ (%pres)) and calculated combined percentage volume of breast ($VxGy_{both\ (calc)}$ (%)) receiving at least 5, 10 and 20 Gray calculated across papers when stating the arm positioning of the patients included.

Dose metric	Category	No. papers	No. regimens	No. plans	Mean (95% CI)
$MBD_{both\ (calc)}$ (Gy)	Arms up	6	11	47	4.3 (2.6 to 5.9)
	Arms akimbo	3	6	99	3.6 (1.4 to 5.8)
	Arms down	7	29	313	3.4 (2.4 to 4.4)
$MBD_{both\ (calc)}$ (%pres)	Arms up	5	9	33	13.8 (6.7 to 20.9)
	Arms akimbo	3	6	99	12.2 (6.5 to 17.9)
	Arms down	7	29	313	10.0 (7.5 to 12.5)
$V5Gy_{both\ (calc)}$ (%)	Arms up	3	5	19	13.2 (1.7 to 24.7)
	Arms akimbo	2	3	24	12.0 (7.6 to 16.4)
	Arms down	5	16	200	18.9 (10.4 to 27.4)
$V10Gy_{both\ (calc)}$ (%)	Arms up	2	4	9	8.5 (-2.4 to 19.3)
	Arms down	3	12	154	18.2 (9.5 to 27.0)
$V20Gy_{both\ (calc)}$ (%)	Arms up	3	6	21	9.3 (1.4 to 17.3)
	Arms akimbo	2	3	24	4.5 (3.7 to 5.4)
	Arms down	3	8	120	4.6 (1.7 to 7.6)

Breathing technique

The majority (30) of papers made no mention of a breathing technique. A similar proportion of papers report female breast dose for patients in free breathing (FB) and deep inspiration breath hold (DIBH) (18 and 14 respectively). 2 papers included patients planned with a mix of FB or DIBH.

5 papers directly compared MBD for FB and DIBH. No difference in $MBD_{both\ (calc)}$ (Gy) was seen between DIBH and FB; 3.0Gy (95%CI 1.9 to 4.1) vs 3.1Gy (95% CI 2.1 to 4.1) (p=1). Insufficient data was found to compare FB and DIBH breast doses at other breast dose levels.

Position on table

Table position was not reported in 29 papers. Only 1 paper directly compared flat and inclined board table positioning, thus there is insufficient data to conclude any difference in breast dose metrics across multiple patient populations between table positions.
